# Long-term Survival and Functional Neurological Outcome in Conscious Hospital Survivors Undergoing Therapeutic Hypothermia

**DOI:** 10.5005/jp-journals-10071-23107

**Published:** 2019-01

**Authors:** Napplika Kongpolprom, Jiraphat Cholkraisuwat

**Affiliations:** 1,2 Division of Pulmonary and Critical Care Medicine, Faculty of Medicine, Chulalongkorn University, Thai Red Cross, Thailand

**Keywords:** Disability rating scale, Functional neurological outcome, Long-term survival, Post-cardiac arrest, Therapeutic hypothermia

## Abstract

**Introduction:**

Therapeutic hypothermia (TH) is the neuroprotective strategy for comatose survivors of cardiac arrest. It improves neurological outcomes at hospital discharge. However, data regarding long-term outcomes are limited. We aimed to study functional ability and survival of the patients after discharge.

**Patients and methods:**

We reviewed data of post-arrest patients undergoing TH in our hospital from 2006 to 2014 and assessed the functional ability of conscious survivors after hospital discharge by using a disability rating scale (DRS). We compared the patients' DRS after discharge with their cerebral performance category (CPC) at hospital discharge. Additionally, we analyzed survival rates at 6 months, 1, 2, and 3 years.

**Results:**

Of 51 patients undergoing TH, 27 survived, and 17 of these were conscious. Approximately 75%, 73%, 71%, and 56% of the hospital survivors were alive at 6 months, 1, 2 and 3 years, respectively. We evaluated the functional ability (DRS) in 15 awake patients*. *The majority of the patients with good performance (CPC1) at discharge returned to normal function or minimal disability (DRS 0-3). Interestingly, although the patients with worse CPC scores at discharge had a greater risk of functional disability and death, a patient with severe disability (CPC3) at discharge fully recovered and was able to return to work later on.

**Conclusion:**

Long-term survival of conscious patients undergoing TH was quite high. The good CPC score at discharge potentially predicted the favorable forthcoming outcome. However, it was difficult to predict the unfavorable long-term outcome from the poor condition at discharge.

**How to cite this article:**

Kongpolprom N, Cholkraisuwat J. Long-term Survival and Functional Neurological Outcome in Conscious Hospital Survivors Undergoing Therapeutic Hypothermia. Indian Journal of Critical Care Medicine, January 2019; 23(1):20-26.

## INTRODUCTION

Therapeutic hypothermia (TH) has been proposed as a standard treatment for comatose survivors of cardiac arrest.^1-3^ Immediate TH should be administered to all cardiac arrest patients without trauma but with the return of spontaneous circulation (ROSC) and a Glasgow coma scale (GCS) <8. This treatment increases both survival rate, and the number of patients with favorable neurological outcome.^4^ Neurological recovery is well-known to improve continuously after discharge. ^5-7^ Glasgow- Pittsburgh cerebral performance category (CPC) score, representing global disability, is better at 6 months and 1 year after discharge than CPC at hospital discharge.^8^ However, CPC score correlates poorly with functional abilities.^9,10^ The score possibly overestimates cognitive function and physical independence. A substantial number of survivors of cardiac arrest subsequently suffer from functional disability-they have decreased capacity for self-care activities and poor quality of life.^11,12^ Some of these survivors were mentally impaired and could not return to work despite mild physical disability.^13^ Disability rating scales determine the ability of an individual to perform daily activities, their ability for self-care, level of dependence, and psychosocial function. The scale score is a good measure of functional neurological outcome.^14,15^ Because data regarding long-term outcomes of these survivors are limited (a reference perhaps a systematic review or meta-analysis), the goals of our study were to evaluate the long-term survival and functional neurological outcomes of survivors previously treated with TH in our hospital who were conscious at discharge. We analyzed factors associated with these functional outcomes.

## PATIENTS AND METHODS

### Patients

We retrospectively reviewed our database of the post-cardiac arrest survivors who underwent TH in 2 medical ICUs and 1 CCU in our tertiary university teaching hospital from 2006 to 2014. The patients were identified from the hospital database using "ICD 10 code I 460-cardiac arrest with successful resuscitation" and "ICD9 code 9961-therapeutic hypothermia" as the diagnosis and intervention.

### Hypothermia Protocol

All comatose survivors from cardiac arrest with GCS <= 8 after ROSC were evaluated for TH. Patients who were eligible for TH were cooled to 32-34° C for 24 hours by external cooling methods with or without internal cooling methods, followed by rewarming. All patients were sedated. Shiverings were treated with extra sedation and neuromuscular blockades. The sedative drugs and neuromuscular blockades were interrupted after completing the rewarming process. Hemodynamic and respiratory parameters were continuously monitored and vasopressors or inotropic drugs were administered to maintain hemodynamic stability. Antiepileptic drugs were prescribed if the patients developed clinical or electrical signs of a seizure.

### Data Collection

Patient data including baseline characteristics, cooling practice, symptoms, signs and clinical outcomes were collected from medical charts and flowsheets. All data were recorded by ICU staff including primary physicians, intensivists, neurologists and critical care nurses.

### Outcome Measures

The CPC at discharge was used to evaluate neurological outcomes. We dichotomized the outcomes as follows: (1a) favorable outcome: CPC1 = good cerebral performance and CPC2 = moderate disability, and (2a) unfavorable outcome: CPC3 = severe disability, CPC4 = vegetative state and CPC5 = brain death or (1b) regained consciousness: CPC1-3 and (2b) unconsciousness: CPC 4-5.

Disability rating scale (DRS) was used to assess functional abilities. ^14^ It consists of 8 items divided into 4 categories; (a) arousability, awareness, and responsivity, including eye-opening, communication ability, and motor response; (b) cognitive ability to handle self-care functions, including feeding, toileting, and grooming; (c) physical dependence upon others, including level of functioning and; (d) psychosocial adaptability for work, including employability. Levels of functional disability are scored as follows; score 0-none; 1-mild; 2 to 3.5- partial; 4 to 6-moderate; 7 to 11-moderately severe; 12 to 16-severe; 17 to 21-extremely severe; 22 to 24-vegetative state; 25 to 29-extreme vegetative state.

All conscious hospital survivors (CPC1-3 at discharge) were evaluated for life or death status and death date on January 31st, 2015 by checking death certificates from the national registry system. We contacted survivors or their relatives by phone or mail and scheduled a follow-up visit. Functional neurological outcome was evaluated by DRS.^15^ Functional disability of patients who were unable to visit was assessed by phone interview or using recorded follow-up data. Survival rates at 6 months, 1, 2 and 3 years after discharge were analyzed. Additionally, we explored factors possibly associated with long-term survival and functional neurological outcomes. The study was approved by the hospital ethics committee.

### Data Analysis

Patient baseline characteristics were described according to types of variables and the normality of their distribution. Continuous variables were reported as mean (SD) or median (interquartile range: IQR). Categorical variables were reported as number or percentages. The factors associated with clinical outcomes were analyzed. Unpaired t-test or Mann-Whitney U-test was used for comparison of continuous variables in the 2 groups. Chi-square or Fisher-exact tests were used to analyze the association between the categorical variables of the two groups. Multivariate analysis was analyzed with logistic regression. We used a two-sided test, and a p-value of less than 0.05 was considered statistically significant. Statistical calculations were performed using the Statistical Package for Social Sciences (SPSS) version 22.

## RESULTS

Fifty-one post-cardiac arrest survivors were treated with TH from 2006 to 2014; 40 (78%) survivors from out-of-hospital and 11 (21.6%) survivors from in-hospital cardiac arrest. The majority of primary cardiac rhythm was non-shockable rhythm (78.4%). The median age of the survivors was 59 years and approximately 57% of them were male. Twenty-seven patients (53%) survived to hospital discharge and 17 of them were conscious: 6, 3 and 8 patients with CPC at discharge 1, 2 and 3, respectively, as in Flowchart 1. Baseline characteristics and clinical data of conscious patients compared with those of unconscious patients are shown in Table 1.

**Graph 1:**
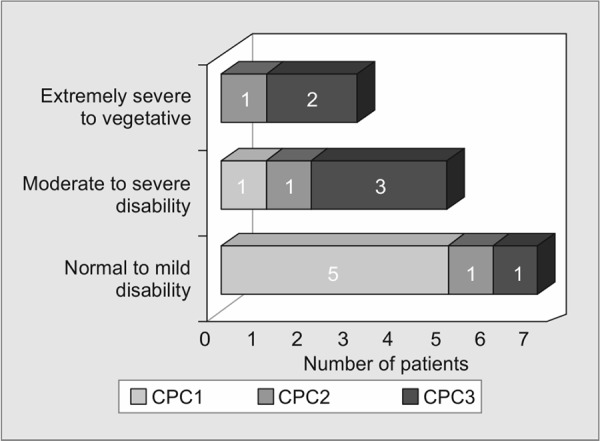
CPC at discharge:CPC1 Vs. CPC2 Vs. CPC3

**Graph 2:**
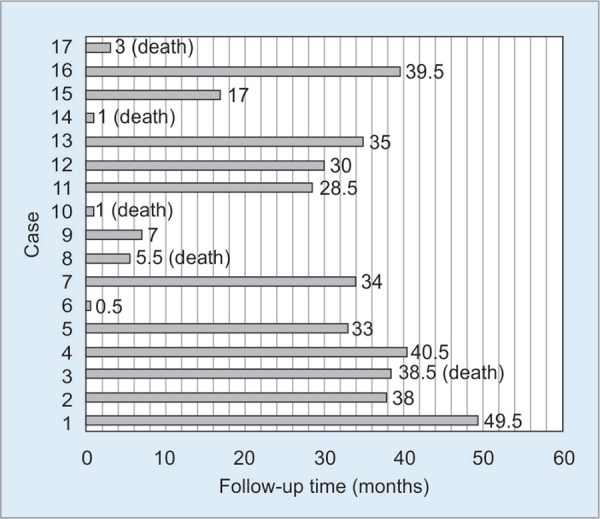
Follow-up duration

**Graph 3:**
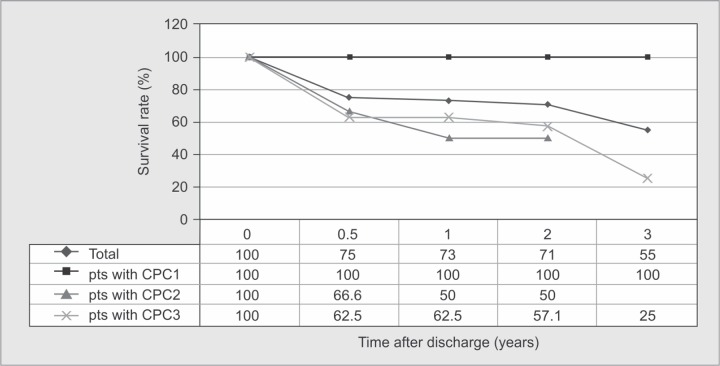
Patients survived at 6 months, 1, 2 and 3 years after discharge

Fifteen awake patients had follow-up data and/or could be contacted and their functional disabilities were assessed. However, the other two survived but were lost to follow-up. Patient DRS scores are shown in Table 2. Most patients with CPC1 at discharge returned to normal function or minimal functional disability (DRS 0-3) except one patient assessed as DRS7 (who later passed away). Interestingly, a patient (No.13) with CPC3 at discharge returned to full physical and functional ability later on as shown in Graph 1. The follow-up duration from hospital discharge to the evaluation date ranged from 0.5 months to 49.5 months as shown in Graph 2. Five patients passed away during the follow-up period, four died within 6 months with extremely severe functional disability (DRS20-26) after discharge, while the other died after 3 years with moderate functional disability (DRS7) after discharge. Approximately 75% (12/16), 73.3% (11/15), 71.4% (10/14) and 55.6% (5/9) of patients survived at 6 months, 1, 2 and 3 years after discharge, respectively, as shown in Graph 3. Noticeably, patients with better CPC at discharge had a greater chance to survive over time.

**Flowchart 1:**
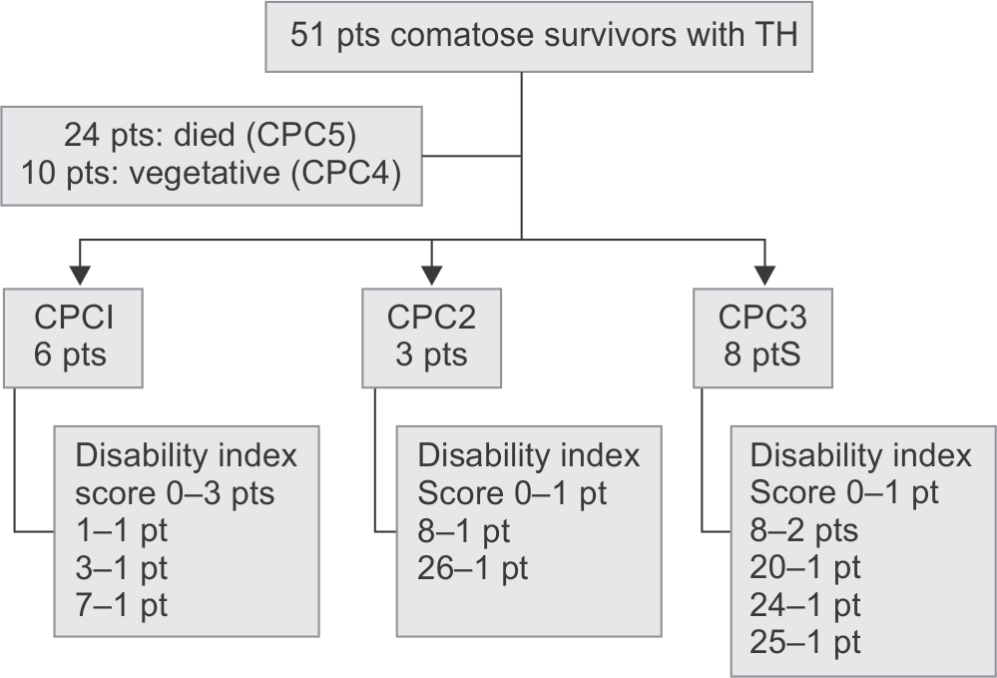
Study population

The factors possibly associated with consciousness at discharge and long term functional disability are shown in Tables 1 and 3. Our univariate analysis demonstrated that gender; male (p = 0.046), free of vasopressors (p = 0.01) and TH protocol completion (p = 0.036) were the factors associated with consciousness at hospital discharge. However, these factors were not statistically significant clinical predictors when adjusted with other confounders (multivariate analysis). In addition, patients with complete recovery (DRS 0) after discharge tended to be younger and had a shorter time from collapse to ROSC.

**Table 1:** The baseline characteristics and clinical data of patients classified with consciousness and unconsciousness or death at hospital discharge

*CPC at hospital discharge*						
	*ALL*	*Conscious CPC 1-3*	*Unconscious CPC 4-5*	*P value*	P value Adjusted*	*Odds ratio# (95%CI)*
n (%)	51	17 (33.3)	34 (66.7)	NS	NS	-
Age, years old, median (IQR)	59	49 (29)	60.5 (21)	NS	NS	-
Gender, n (%)						
Male	29 (56.9)	13 (76.5)	16 (47.1)	0.046	0.146	0.27
Female	22 (43.1)	4 (23.5)	18 (52.9)			(0.07 to 1.01)
Location of cardiac arrest,n (%)						
Out-of hospital	40 (78.4)	15 (88.2)	25 (73.5)	NS	NS	2.7
In-hospital	11 (21.6)	2 (11.8)	9 (26.5)			( 0.5 to 14.2)
Primary cardiac arrest rhythm,n (%)						
Shockable	11 (21.6)	5 (29.4)	6 (17.6)	NS	NS	1.94
Nonshockable	40 (78.4)	12 (70.6)	28 (82.4)			( 0.5 to 7.62)
Cause of cardiac arrest				-	-	-
Arrythmia	9	2	7			
MI	16	7	9			
Electrical injury	2	2	0			
Brugada	5	2	3			
Others	19	4	15			
Delayed CPR, minutes, median (IQR)	10 (15)	10 (8.5)	10 (16.5)	NS	NS	-
CPR duration, minutes, median (IQR)	15(10)	14 (9.5)	18 (21.75)	NS	NS	-
CPR number, median (IQR)	1 (0)	1 (0)	1 (0)	NS	NS	-
Collapse to ROSC, minutes, median (IQR)	26 (18)	24 (8)	29.5 (26)	NS	NS	-
ROSC to initiate TH, hours, median (IQR)	5 (3)	5 (2.34)	4.17(5.63)	NS	NS	-
Time from TH induction to achieved target, hours, median (IQR)	4 (3)	4 (4)	3.75 (3.38)	NS	NS	-
ROSC to achieved target tempertature, hours, median (IQR)	10.5 (6)	11 (5)	9.5 (5.88)	NS	NS	-
Target temperature achievement, n (%)	43 (84.3)	15 (88.2)	28 (82.4)	NS	NS	0.62 (0.11-3.47)
ROSC to target temperature < 6 hrs, n (%)	4	0(0)	4 (14.8)	NS	NS	5.16 (0.26-101.7 )
Need of vasopressor, n (%)	25 (46)	4 (23.5)	21 (61.8)	0.01	0.055	5.25 (1.41-19.59 )
Protocol completion, n (%)	36 (70.6)	16 (88.2)	23(61.8)	0.036	0.465	0.13 (0.02-1.12)
Postrewarm pyrexia, n (%)	20 (39.2)	6 (35.3)	14 (41.2)	NS	NS	1.28 (0.38-4.29)
LOS, days, median (IQR)	13 (20)	13 (19.5)	9.5 (21)	NS	NS	-

* Variables were adjusted for gender, need of vasopressor, protocol completion, ROSC to target temperature < 6 hours, ROSC to initiate TH < 6 hours, target temperature achievement and postrewarm pyrexia.

# Odds ratio; compare between CPC 1-3 *vs. *CPC 4-5

**Table 2:** Disability rating scores of conscious hospital survivors undergoing therapeutic hypothermia

*No*	*CPC at D/C*	*Alive VS Dead Status / Cause of death*	*Date of DRS evaluation*	*1*			*2*			*3*	*4*	Total score**
				*A*	*B*	*C*	*D*	*E*	*F*	*G*	*H*	
1.	1	Alive	10 May 2011	0	0	0	0	0	0	1	0	1
2.	1	Alive	19 Dec 2011	0	0	0	0	0	0	0	0	0
3.	1	Dead / HIV	2 Jul 2009	0	0	0	1	1	1	2	2	7
4.	1	Alive	1 Sep 2011	0	0	0	0	0	1	1	1	3
5.	1	Alive	20 Jan 2015	0	0	0	0	0	0	0	0	0
6.	1	Alive	4 Jan 2015	0	0	0	0	0	0	0	0	0
7.	2	Alive	1 Apr 2012	0	0	1	1	1	1	2	2	8
8.	2	Dead / multiple infections & aspiration	16 Sep 2012	3	4	3	3	3	3	4	3	26
9.	2	Alive	23 Dec 2014	0	0	0	0	0	0	0	0	0
10.	3	Dead / NA	1 Feb 2010	2	4	3	3	3	3	4	3	25
11.	3	Alive	NA	Could not be contacted								NA
12.	3	Alive	1 Apr 2013	1	1	0	0	1	1	1	3	8
13.	3	Alive	15 Aug 2014	0	0	0	0	0	0	0	0	0
14.	3	Dead / COPD	1 Feb 2010	3	3	2	3	3	3	4	3	24
15.	3	Alive	NA	Could not be contacted								NA
16.	3	Alive	2 Dec 2014	0	0	0	1	1	1	2	3	8
17.	3	Dead / NA	4 Jan 2009	2	1	1	3	3	3	4	3	20

* Disability rating scale: 1) Arousability awareness and responsivity: A-Eye opening; B-Communication ability; C-Motor response, 2) Cognitive ability for self care activities: D-Feeding; E-Toileting; F-Grooming, 3) Dependence on others: G-Level of functioning, 4) Psychosocial adaptability: H-Employability

** Level of disability: Score 0-None; 1-Mild; 2 to 3.5-Partial; 4 to 6-Moderate; 7 to 11-Moderately severe; 12 to 16-Severe; 17 to 21- Extremely severe; 22 to 24-Vegetative state; 25 to 29-Extreme vegetative state

NA; not available

## DISCUSSION

Therapeutic hypothermia improved survival and short term neurological recovery of post-cardiac arrest patients.^4^ Our study showed that during the 8 year study period, only 51 comatose survivors received TH despite hospital TH protocol implementation. There were several possible explanations for this low rate of TH provision. Firstly, most comatose survivors had a prolonged period of over 30 minutes from collapse to ROSC. Secondly, some patients suffered from chronic illness with poor performance status before cardiac arrest. Furthermore, some primary physicians were still unaware of this treatment. Lastly and most importantly, a limited number of ICU beds precluded patients from this intervention.

Our study showed approximately 53% (27/51) of comatose survivors treated with TH survived to hospital discharge, but only 63% (17/27) became conscious at discharge. The proportion of patients with favorable neurological outcome was lower than other studies due to the fact that our hospital protocol included patients with in-hospital cardiac arrest, non-shockable rhythm, and prolonged CPR duration, all having greater risks of poor outcome.^4,5,16-18^

**Table 3:** Possible factors associated with long-term functional disability*

	*Normal*	*Mild to moderate disability*	*Severe disability to vegetative*	*Any disability*		
		*DRS Score*	*DRS Score*	*DRS Score*		
*Disability index*	*DRS Score 0* *n = 5*	*1–11* *n = 6*	*12–29* *n = 4*	*1–29* *n = 10*	*Odds ratio#*	*95% CI*
Age, years old, median (IQR)	39 (50)	60 (25)	44 (NA)	57 (24)	-	-
Delayed CPR, minutes, median (IQR)	9.5 (14.5)	9 (2.5)	14 (NA)	10 (4)	-	-
CPR duration, minutes, median (IQR)	12 (13)	14 (9.5)	12.5 (NA)	14 (6)	-	-
CPR number, median (IQR)	1 (1)	1 (1)	1 (NA)	1 (0)	-	-
Collapse to ROSC, minutes, median (IQR)	22.5 (4.5)	22 (10.5)	26.5 (NA)	25 (7)	-	-
ROSC to initiate TH, hrs, median (IQR)	5.75 (3.38)	6 (3)	5.5 (NA)	6 (2)	-	-
Time from TH induction to achieved target, hrs, median (IQR)	5 (7.25)	4 (3)	14 (NA)	4 (4)	-	-
ROSC to achieved target tempertature, hrs, median (IQR)	11.75 (5.88)	11 (5.5)	19.5 (NA)	12 (6)	-	-
Target temperature achievement, n (%)	5 (100)	5 (83.3)	3 (75)	8 (80)	0.31	0.01-7.74
ROSC to target temperature < 6 hrs, n (%)	0 (0)	0 (0)	0 (0)	0 (0)	0.52	0.01-30.17
Cooling duration, hrs, median(IQR)	23.5 (10.75)	28 (5)	27.5 (NA)	28 (6)	-	-
Need of vasopressor, n (%)	1 (20)	1 (16.7)	1 (25)	2 (20)	1	0.07-14.64
Protocol completion, n (%)	4 (80)	5 (83.3)	4 (100)	9 (90)	0.44	0.02-9.03
Postrewarm pyrexia, n (%)	1 (20)	2 (33.3)	1 (25)	3 (30)	1.71	0.13-22.51

*The number of patient was insufficient to analyze statistical significance.

# Odds ratio; compare patients with and without any disability (mild to severe disability *vs. *normal)

Of 17 conscious hospital survivors, five patients had a full functional recovery later and surprisingly, one returned from severe disability at discharge. Similar to other studies, it was difficult to predict long-term outcome from the condition at discharge.^9,19^ Hsu et al. reported that CPC at discharge poorly correlated with quality of life at least 6 months after discharge.^20^ Moreover, although patients with worse CPC at discharge had a higher risk to develop unfavorable outcome over time and needed a longer time to recovery, some of them possibly returned to their baseline performance. Thus, for patients with CPC3 at discharge, physicians should not classify them as poor status or withdraw life-supporting treatment.

Furthermore, except for a recently discharged patient, all of our patients with CPC 1 at discharge survived longer than 2.5 years, although half of them were mildly dependent (DRS1-3) and one patient died at 38.5 months after discharge due to his underlying disease. Interestingly, regardless of CPC at discharge, conscious patients who survived beyond 6 months returned to their abilities with at the least partial dependence (DRS ≤8). Additionally, we evaluated clinical factors determining long-term functional recovery and found that patients with full recovery tended to be younger and had a shorter time from collapse to CPR initiation, shorter time from collapse to ROSC and shorter time from ROSC to achieving targeted temperature.

Survival rates of our patients at 6 months and 1 year after discharge were comparable to other studies.^13^ However, we could not evaluate the proportion of patients who finally recovered to CPC1 at 6 months and 1 year after discharge due to limited data. Hsu et al. also reported that patients with worse CPC scores were associated with higher risk of death. ^20^ In this study, patients with CPC 3 had a hazard ratio of death of 3.62 (95% CI 1.06, 12.35), compared with patients with CPC1. Similar to this finding, the survival rate of our patients with CPC3 was lower than those with CPC1.

Most studies on long-term outcomes of post-arrest survivors undergoing TH generally demonstrated survival rates and a favorable outcome, defined as CPC 1 or 2, or a good outcome, defined as discharge home or to a rehabilitation facility, despite the fact that functional dependence and impaired cognitive abilities commonly appeared.

In our study, we demonstrated patients' ability to do self-care activities by DRS score at a single point of time. The score was more sensitive to detect the functional disability than CPC. The median time to the assessment of the DRS score was 30 months after hospital discharge. At this time point, 1-9 patients had a motor problem, while 5 of 9 patients had mildly impaired cognitive performance including feeding, toileting, and grooming. Besides, only 3 of 9 patients returned to work. However, psychological problems and other cognitive functions such as memory disturbance, dementia were not demonstrated in the study.

Additionally, there were recent studies demonstrating other functional outcomes in post-arrest survivors. Larsson et al. serially measured self-reported health-related quality of life (QOL) by the questionnaires EQ-5D and SF-12, anxiety, and depression of post-arrest survivors undergoing TH at hospital discharge, 1 month, and 6 months. They found that mobility problems reduced from 54% at discharge to 31% at 6 months. QOL for the physical and mental components improved over the time while anxiety/depression did not improve.^7^

Similarly, Raina et al. showed functional recovery continued over the time, but depressive symptoms were common at 1 year after discharge.^9^ Furthermore, Smith et al. published the largest study which assessed the quality of life of patients with a history of out-of-hospital cardiac arrest. They found the survivors had acceptable QOL at 1 year, assessed by EQ-5D index.^13^

For memory disturbance, Jennifer et al. assessed the disability by using the telephone interview for cognitive status, modified (TICS-m) at the median time of 20 months after hospital discharge in post-arrest patients and found that 22 of 56 patients (40%) had cognitive impairment (TICS-m <32) and 10 of 22 patients with cognitive impairment (45%) had low TICS-m scores (TICS-m ≤27), consistent with dementia.^21^

Importantly, the data confirmed that neurological recovery after cardiac arrest continued for several months. However, some patients suffered from functional disabilities and psychological stress after hospital discharge. Therefore, multidisciplinary assessment for functional abilities, QOL, and psychiatric morbidities should be provided to all post-arrest survivors.

Our study had several limitations. Firstly, the sample size was small. Secondly, time points after discharge for neurological function assessment in each patient were not consistent. Data were recorded at different times based on either first availability depending on the last recorded data or on dates we were able to contact patients or their relatives. Therefore, disability rating scores (DRS) were not the current scores in some patients.

Moreover, the length of follow-up time was also variable (0.5-49.5 months), which expected the accuracy of survival rate at second and third years due to the small number of patients.

Furthermore, there were insufficient data of serial neurological examinations to assess how long patients needed to improve physical and functional ability. Additionally, due to the retrospective design of the study, some information was missed and we did not know the baseline functional performance of patients before cardiac arrest. Lastly, we evaluated the general functional ability to do daily activities, not including some cognitive functions such as memory, cognitive speed, and visuospatial performance.

## CONCLUSION

Overall, long-term survival rate of awake patients was quite high-approximately 70 to 75% in the first 2 years after discharge. Survivors with better CPC scores at discharge had greater functional capability to perform daily activities and lower hazards of death. The possible associated factors for long-term neurological recovery were age, time from collapse to CPR initiation, time from collapse to ROSC and time from ROSC to achieving targeted temperature.
